# Subcellular distribution of glutathione and its dynamic changes under oxidative stress in the yeast *Saccharomyces cerevisiae*

**DOI:** 10.1111/j.1567-1364.2011.00753.x

**Published:** 2011-09-27

**Authors:** Bernd Zechmann, Liang-Chun Liou, Barbara E Koffler, Lucija Horvat, Ana Tomašić, Hrvoje Fulgosi, Zhaojie Zhang

**Affiliations:** 1 Institute of Plant Sciences, University of GrazGraz, Austria; 2 Institute for Electron Microscopy and Fine Structure Research, Graz University of TechnologyGraz, Austria; 3 Department of Zoology and Physiology, University of WyomingLaramie, WY, USA; 4 Department of Molecular Biology, Ruer Bošković InstituteZagreb, Croatia

**Keywords:** glutathione, hydrogen peroxide, immunogold labeling, mitochondria, oxidative stress

## Abstract

Glutathione is an important antioxidant in most prokaryotes and eukaryotes. It detoxifies reactive oxygen species and is also involved in the modulation of gene expression, in redox signaling, and in the regulation of enzymatic activities. In this study, the subcellular distribution of glutathione was studied in *Saccharomyces cerevisiae* by quantitative immunoelectron microscopy. Highest glutathione contents were detected in mitochondria and subsequently in the cytosol, nuclei, cell walls, and vacuoles. The induction of oxidative stress by hydrogen peroxide (H_2_O_2_) led to changes in glutathione-specific labeling. Three cell types were identified. Cell types I and II contained more glutathione than control cells. Cell type II differed from cell type I in showing a decrease in glutathione-specific labeling solely in mitochondria. Cell type III contained much less glutathione contents than the control and showed the strongest decrease in mitochondria, suggesting that high and stable levels of glutathione in mitochondria are important for the protection and survival of the cells during oxidative stress. Additionally, large amounts of glutathione were relocated and stored in vacuoles in cell type III, suggesting the importance of the sequestration of glutathione in vacuoles under oxidative stress.

## Introduction

The formation of reactive oxygen species (ROS) is inevitable in aerobic environments owing to the reactive nature of molecular oxygen. ROS such as superoxide anion radicals, hydrogen peroxide (H_2_O_2_), and hydroxyl radicals are highly reactive chemicals, which can lead to the destruction of biologic membranes, proteins, RNA, and DNA leading to mutations, cancer, or cell death (cf. Halliwell & Gutteridge, 1984; Halliwell, 1987, 1995; Tausz, 2001; Dawes, 2004; Van Breusegem & Dat, 2006; Herrero *et al*., 2008; López-Mirabal & Winther, 2008; Perrone *et al*., 2008). In *Saccharomyces cerevisiae*, ROS can be produced, for example, by electron leakage from the mitochondrial respiratory chain during aerobic respiration (Bouveris & Cadenas, 1982; Gardner & Fridovich, 1991), during the oxidation of fatty acids, the detoxification of superoxide anions, and various oxidase reactions in case of H_2_O_2_ (Tu *et al*., 2000; Tu & Weissman, 2004; Gross *et al*., 2006; Halliwell & Gutteridge, 2007), and during Fenton and Haber–Weiss reactions in case of hydroxyl radicals (Halliwell & Aruoma, 1991; Perrone *et al*., 2008).

Glutathione (γ-glutamyl-cysteinyl-glycine) is one of the most abundant and important antioxidants that detoxifies ROS inside the eukaryotic cell. It is present in all eukaryotic cells in its reduced (GSH) and oxidized form (GSSG). ROS are detoxified by the oxidation of the sulfhydryl group of GSH either directly or through the ascorbate-glutathione cycle (Perrone *et al*., 2008; Foyer & Noctor, 2009). During oxidative stress, large amounts of GSSG can be formed, which are most probably relocated and stored in vacuoles (Queval *et al*., 2011) or reduced to GSH by glutathione reductase. Besides the importance of glutathione as an antioxidant, it is also involved in redox signaling, regulating gene expression and enzymatic activities (Foyer & Noctor, 2009). In *S. cerevisiae*, glutathione also plays important roles during the detoxification of cadmium (Mendoza-Cózatl *et al*., 2005) and the protection of proteins from oxidation by a process called glutathionylation, which is the formation of mixed disulfides between a protein thiol and glutathione (Giustarini *et al*., 2004; Dixon *et al*., 2005; Hurd *et al*., 2005; Iversen *et al*., 2010). Glutathione biosynthesis in *S. cerevisiae* takes place in two ATP-depending steps. In the first step, cysteine is linked with glutamate by γ-glutamylcysteine synthetase (encoded by *GSH1*) to form γ-glutamylcysteine. In the second step, glycine is added to this intermediate product by glutathione synthetase (encoded by *GSH2*) to form the final product glutathione (Grant, 2001; Mendoza-Cózatl *et al*., 2005; Perrone *et al*., 2008).

Mitochondria are the main source of ROS production, via oxidative phosphorylation. They are also the main organelle for the detoxification of ROS. Not surprisingly, it has been reported that, in plants, glutathione concentrations are found to be highest in mitochondria, although other subcellular compartments also contain certain levels of glutathione (Zechmann & Müller, 2010). The compartment-specific distribution of glutathione is most studied using quantitative immunoelectron microscopy, which allows simultaneous detection and quantification at high resolution (Hjelle *et al*., 1994; Huster *et al*., 1998; Ong *et al*., 2000; Zechmann *et al*., 2008; Zechmann & Müller, 2010).

In this study, quantitative immunoelectron microscopy was employed to investigate the subcellular distribution of glutathione in *S. cerevisiae*. We also aimed to study the dynamic changes of the glutathione distribution under conditions of glutathione synthesis deficiency (knockout mutants of *GSH1* and *GSH2*) and oxidative stress. This study confirms mitochondria as the hotspot for glutathione accumulation in eukaryotes and emphasizes the importance of high glutathione levels in mitochondria in the protection against oxidative stress.

## Materials and methods

### Yeast strains and culture condition

Yeast strain BY4742 and derived deletion strains of *GSH1* (*gsh1Δ*) and *GSH2* (*gsh2Δ*) were purchased from Open-Biosystems (Huntsville, AL). Wild-type cells were all grown at 30 °C in YPD (1% yeast extract, 2% peptone, 2% glucose) liquid medium or synthetic complete medium without glutathione (SC^−GSH^). *gsh1Δ* and *gsh2Δ* strains were grown on YPD only as growth was not promoted on medium without GSH. H_2_O_2_ treatment was performed by adding 5 mM H_2_O_2_ to YPD medium containing cells grown to early log phase (5 × 10^6^ cells mL^−1^). Cells remained in the medium for either 30 or 60 min before they were used for different experiments.

### Electron microscopical studies

#### Fixation and embedding

For immunogold labeling, cells were grown to early log phase (5 × 10^6^ cells mL^−1^) and then harvested by gentle centrifugation. The harvested cells were fixed with 4% formaldehyde and 0.25% glutaraldehyde in 40 mM phosphate buffer (pH 6.7), containing 1 mM MgCl_2_ and 1 mM EGTA for 1 h at room temperature. Cells were washed with phosphate buffer and incubated in 1% sodium metaperiodate for 15 min and then in 50 mM ammonium chloride for 15 min. Cells were dehydrated with graded ethanol and embedded in LR White resin (Electron Microscopy Sciences, Hatfield, PA). For ultrastructural observation, cells were harvested by gentle centrifugation, washed in phosphate-buffered saline (PBS; pH 7.2), resuspended in 2.5% (v/v) glutaraldehyde in PBS, and fixed for 40 min at room temperature. Cells were further fixed by freshly prepared 2% potassium permanganate in water for 1 h at room temperature. Fixed cells were dehydrated with 30%, 50%, 75%, 85%, 95%, and 100% ethanol, transitioned with propylene oxide, and embedded with Spurr resin (Electron Microscopy Sciences).

#### Cytohistochemical determination of glutathione

Immunogold labeling of glutathione was performed with ultrathin sections on coated nickel grids with the automated immunogold labeling system Leica EM IGL (Leica Microsystems, Vienna, Austria) according to Zechmann *et al*. (2008) and Zechmann & Müller (2010). For cytohistochemical analysis, samples were blocked with 2% bovine serum albumin in PBS (pH 7.2) for 20 min at room temperature. The samples were then treated with the primary antibody against glutathione [antiglutathione rabbit polyclonal immunoglobulinG (IgG); Millipore Corp., Billerica, MA] diluted 1 : 50 in PBS for 2 h. After short rinses in PBS (three times for 5 min), the samples were incubated with a 10-nm gold-conjugated secondary antibody (goat anti-rabbit IgG; British BioCell International, Cardiff, UK) diluted 1 : 50 in PBS for 90 min. After short washes in PBS (three times for 5 min) and distilled water (two times for 5 min), labeled grids were either immediately observed under a Philips CM10 transmission electron microscope or poststained with uranyl acetate (2% dissolved in aqua bidest) for 15 s. Poststaining with uranyl acetate was applied to facilitate the distinction of different cell structures enabling a clearer identification of the investigated organelles. Negative controls were treated either with (i) gold-conjugated secondary antibody (goat anti-rabbit IgG) without prior incubation of the section with the primary antibody, (ii) nonspecific secondary antibody (goat anti-mouse IgG), (iii) preimmune serum instead of the primary antibody, and (iv) primary antibodies preadsorbed with an excess of either GSH or GSSG for 2 h at RT prior to labeling of the sections. For the latter, a solution containing 10 mM of GSH or GSSG was incubated with or without 0.5% glutardialdehyde for 1 h. The excess of glutardialdehyde was then saturated by incubating for 30 min in a solution of 1% (w/v) BSA. The resulting solutions were used to saturate the glutathione antibody for 2 h prior to its use in the immunogold labeling procedure described earlier.

#### Quantitative analysis of immunogold labeling

Micrographs of randomly photographed immunogold-labeled sections were digitized, and gold particles were counted automatically in different cell compartments using the software package cell d with the particle analysis tool (Olympus, Life and Material Science Europa GmbH, Hamburg, Germany). A minimum of 50 sectioned cells from two independent experiments were analyzed for gold particle density. The obtained data were presented as the number of gold particles per μm^2^. For statistical analyses, either the Mann–Whitney *U*-test or the nonparametric Kruskal–Wallis test followed by a *post hoc* comparison according to Conover was used. *P* < 0.05 was considered as significant (Bortz *et al*., 2008).

### Biochemical determination of glutathione contents

Wild-type yeast cells were grown to log phase on YPD medium and on SC^−GSH^. After H_2_O_2_ treatment, performed by adding 5 mM H_2_O_2_ for 60 min to cells on YPD and SC^−GSH^, respectively, treated cells and control cells were harvested by gentle centrifugation. Supernatant, containing extracellular fluid, was collected and stored on ice, while cell pellet was further processed to obtain intracellular fluid. Pellet was washed three times with ice-cold water, and each time centrifuged at 1000 ***g***, for 3 min, at +4 °C. Subsequently, cells were lysed by sonication and centrifuged at 25 000 ***g***, for 15 min, at +4 °C. Supernatant, containing intracellular fluid, was collected. The amount of total glutathione was determined fluorometrically in the form of GSH using BioVision Glutathione Assay kit (BioVision, Mountain View, CA) according to the manufacturer's directions for both extracellular and intracellular fluids.

### Determination of cell survival rates and cell death

#### Spot assays

Cells were harvested by centrifugation, washed twice with water, and then resuspended in water. The cell density was normalized to a cell concentration = 1 × 10^7^ cells mL^−1^. A fivefold serial dilution of this culture was made, and 4 μL of each dilution was spotted onto YPD solid medium for 3 days.

#### Rhodamine-phalloidin and DAPI staining

For visualization of actin filaments, cells were fixed with 4% formaldehyde in PBS buffer for 1 h. After washing three times with PBS buffer, fixed cells were resuspended in 1 mL PBS containing 1% Triton X-100 and incubated at room temperature for 3 min for cell permeabilization. Permeabilized cells were washed with PBS buffer and resuspended in 20 μL rhodamine-phalloidin (Invitrogen, Carlsbad, CA) (final concentration = 160 nM) for 1 h. For visualization of DNA, cells were stained with 0.5–1 μg mL^−1^ of 4,6-diamidino-2-phenylindole (DAPI), mounted on a glass slide, and viewed under a Zeiss 710 laser scanning confocal microscope.

## Results

### Electron microscopical studies

#### Glutathione distribution in wild-type cells under normal growth condition

**Fig. 1:**
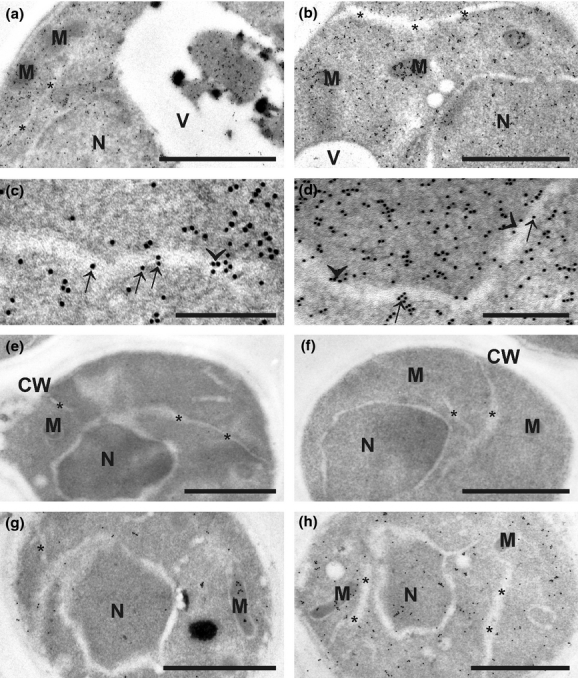
Transmission electron micrographs showing the subcellular distribution of glutathione in *Saccharomyces cerevisiae* grown on YPD (a, e–h) and SC^−GSH^ media (b–d). Gold particles bound to glutathione could be found in mitochondria (M), nuclei (N), vacuoles (V), the ER (asterisks), and the cytosol of *S. cerevisiae* grown on YPD and SC^−GSH^ media. Note that *S. cerevisiae* grown on SC^−GSH^ medium (b) contain higher amounts of gold particles bound to glutathione in mitochondria than cells grown on YPD medium (a). Additionally, gold particles bound to glutathione could be observed in vacuoles of *S. cerevisiae* grown on YPD medium (a). Gold particles could also be found along the membranes (arrows in c) and also inside the lumen (arrowheads in c) of the ER in cells treated with 5 mM H_2_O_2_ for 60 min. Gold particles were also present in the perinuclear space (arrows in d) and along the inner membrane of the nucleus (arrowheads in d). No or only very few gold particles were detected in *S. cerevisiae* after sections were incubated with the primary antibody preadsorbed with an excess of oxidized glutathione (e) and with preimmune serum instead of the primary antibody (f). The glutathione-deficient mutants *gsh1Δ* (g) and *gsh2Δ* (h) contained markedly lower levels of gold particles when compared to the controls. CW, cell walls. Bars = 1 μm in a, b, e–h and 0.25 μm in c, d.

**Fig. 2:**
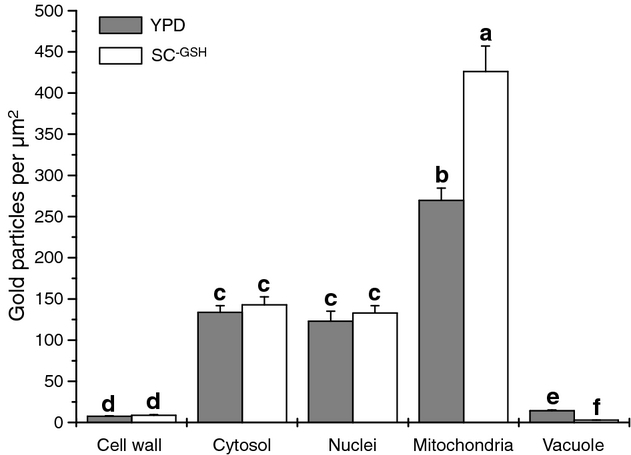
Subcellular distribution of glutathione in *Saccharomyces cerevisiae*. Graph shows the compartment-specific distribution of gold particles bound to glutathione in μm^2^ (means with SE) in *S. cerevisiae* grown on YPD and SC^−GSH^ media. Significant differences between the samples are indicated by different lower case letters; samples that are significantly different from each other have no letter in common. *P* < 0.05 was regarded significant, analyzed by the Kruskal–Wallis test, and followed by *post hoc* comparison according to Conover. *n* ≥ 50 cells per growth medium.

Immunogold labeling showed that, under normal growth condition (YPD liquid medium), glutathione is distributed in all identifiable subcellular compartments, including cell wall, cytosol, nucleus, mitochondria, and vacuoles (Fig. 1a). The distribution, however, is uneven among different compartments. Quantitative analysis of more than 50 cells revealed that the highest labeling density (gold particles per μm^2^) was in mitochondria, whereas the lowest density was in cell wall and vacuoles (Fig. 2). To examine whether glutathione is synthesized by cells *de novo*, or absorbed from the culture medium, yeast cells were cultured in a synthetic complete medium lacking GSH (SC^−GSH^). Based on whole-cell labeling, no significant difference of the labeling density was observed between cells growing on YPD or SC^−GSH^ (Fig. 1a, b and 2), suggesting that glutathione was primarily synthesized by cells internally. Interestingly, significantly higher glutathione-specific labeling in mitochondria was observed when compared to cells grown in YPD medium (Fig. 2). Glutathione was also frequently observed at the membranes and within the lumen of the endoplasmic reticulum (ER) and also in the perinuclear space (Fig. 1c and d). Preadsorption of the glutathione antibody with GSSG reduced labeling density to background level (Fig. 1e). Additionally, gold particles were absent on sections after they were treated with preimmune serum instead of the primary antibody (Fig. 1f).

### Glutathione distribution in glutathione synthesis–deficient mutants *gsh1Δ* and *gsh2Δ*

**Fig. 3:**
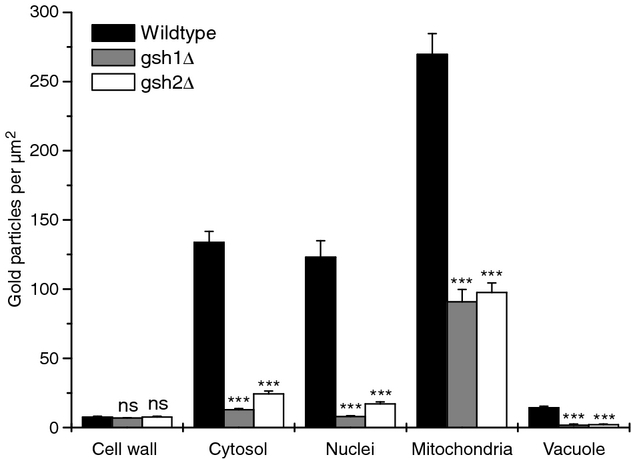
Subcellular distribution of glutathione in the glutathione-deficient mutants *gsh1Δ* and *gsh2Δ*. Graph shows the compartment-specific distribution of gold particles bound to glutathione in μm^2^ (means with SE) in *Saccharomyces cerevisiae* grown on YPD medium. Significant differences between the mutants and the wild type were calculated with the Mann–Whitney *U*-test. ns if *P* > 0.05, ***P < 0.001. *n* ≥ 50 cells per mutant.

Deletion of genes involved in glutathione biosynthesis, *GSH1* or *GSH2*, greatly reduced the glutathione-specific labeling in the cytosol (−90%, −82%), nuclei (−94%, −86%), mitochondria (−66%, −64%), and vacuoles (−86%, −83%; Figs 1g, h and 3), further confirming that glutathione was primarily synthesized by cells, rather than absorbed from the medium. Based on the whole-cell labeling, the *gsh1Δ* and *gsh2Δ* contained only 11% and 20% of glutathione-specific labeling when compared to the wild type. This result also suggests that the deletion of *GSH2* has a lesser effect than *GSH1* on glutathione synthesis. The *gsh1Δ* and *gsh2Δ* mutants showed decreased glutathione-specific labeling in all cell compartments except cell wall (Fig. 3), but the labeling intensity showed a similar pattern compared with wild type (i.e. the mitochondria have the highest, while the cell wall the lowest labeling intensity).

### Glutathione distribution in wild-type cells treated with hydrogen peroxide (H_2_O_2_)

**Fig. 4:**
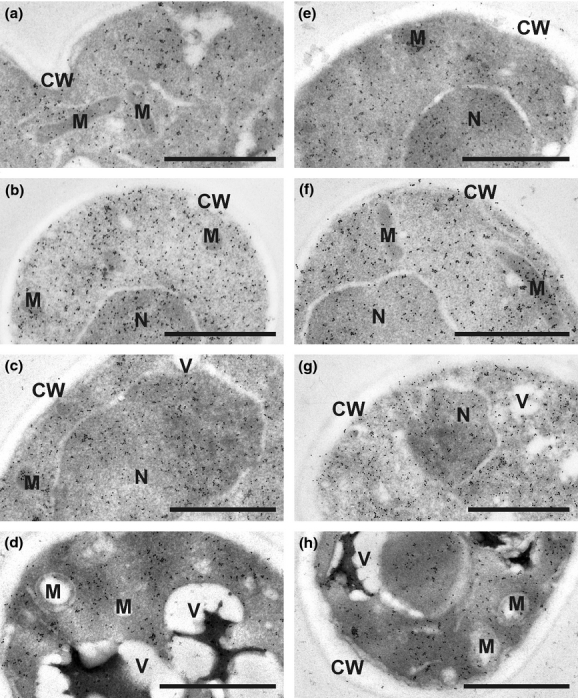
Transmission electron micrographs showing the subcellular distribution of glutathione in *Saccharomyces cerevisiae* grown on YPD (a–d) and SC^−GSH^ media (e–h). Control cells (a, e) show glutathione labeling in mitochondria (M), nuclei (N), the cytosol, and cell walls (CW). Three different cell types could be differentiated after cells were treated with 5 mM H_2_O_2_ for 60 min. Cell type I (b, f) shows highest glutathione labeling density followed by cell type II (c, g), which still contains more gold particles bound to glutathione than the wild type. Cell type III (d, h) shows lowest glutathione labeling density and typical signs of cell death, for example increased vacuolation and swollen mitochondria. Additionally, cell type III contained black inclusions in vacuoles, which contained large amounts of gold particles bound to glutathione. Bars = 1 μm.

**Fig. 5:**
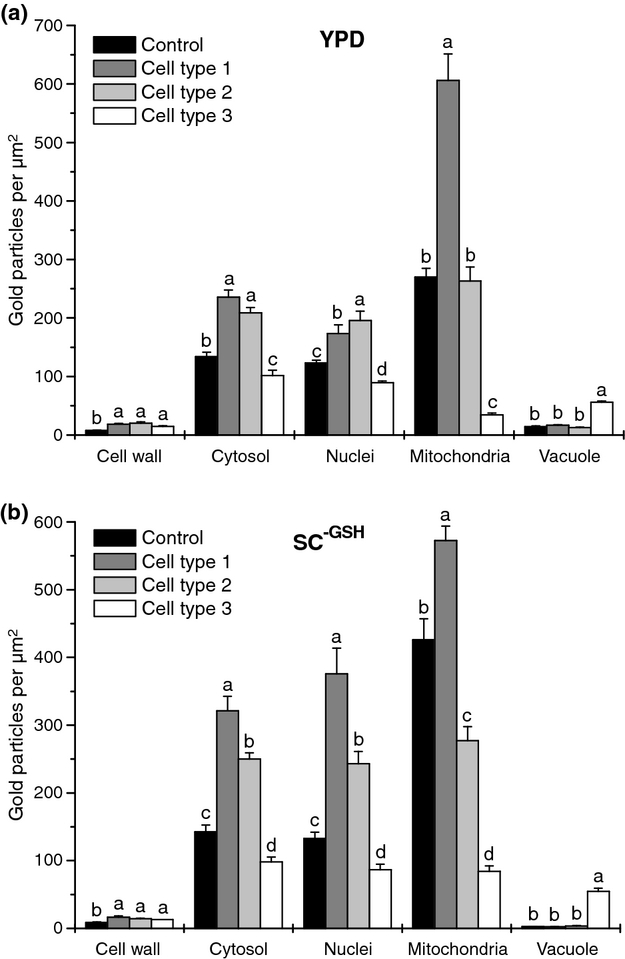
Subcellular distribution of glutathione in *Saccharomyces cerevisiae* after the treatment with 5 mM H_2_O_2_ for 60 min. Graphs show the compartment-specific distribution of gold particles bound to glutathione per μm^2^ (means with SE) in *S. cerevisiae* grown on YPD and SC^−GSH^ media with the addition of H_2_O_2_. Three different cell types could be distinguished after H_2_O_2_ treatment when cells were compared with untreated cells (control): cell type I showed increased amounts of gold particles bound to glutathione in all cell compartments; cell type II showed elevated gold particle density in all cell compartments except mitochondria, and cell type III showed decreased levels of glutathione in most cell compartment except cell walls and vacuoles. Significant differences were calculated for each compartment, and differences between the samples are indicated by different lower case letters; samples that are significantly different from each other have no letter in common. *P* < 0.05 was regarded significant, analyzed by the Kruskal–Wallis test, and followed by *post hoc* comparison according to Conover. *n* ≥ 50 cells per growth medium.

**Fig. 6:**
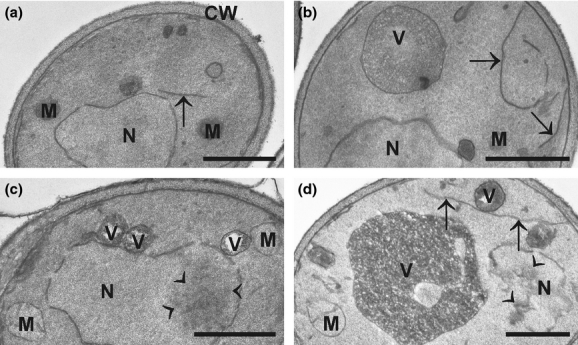
Transmission electron micrographs showing the ultrastructure of *Saccharomyces cerevisiae* grown on YPD media without (a) and with (b, c, d) the addition of 5 mM H_2_O_2_ for 60 min. The control cell (a) contains a large nucleus (N), mitochondria (M), and ER (arrows) within the dense cytosol, which is surrounded by a cell wall (CW). Two different cell types could be distinguished in cells grown on media with H_2_O_2_. Image (b) shows a cell that corresponds to cell types I and II according to gold particle density documented in Figs 4 and 5. This cell type looks similar to the control with a large nucleus (N), mitochondria (M), a central vacuole (V), and ER (arrows) in the dense cytosol. Images (c, d) show cells that correspond to cell type III according to gold particle density documented in Figs 4 and 5. These cells contain numerous vacuoles (V) filled with heavily granulated electron-dense material, swollen mitochondria (M), nucleus (N) with partly condensed chromatin (arrowheads), and ER (arrows). Bars = 1 μm.

**Fig. 7:**
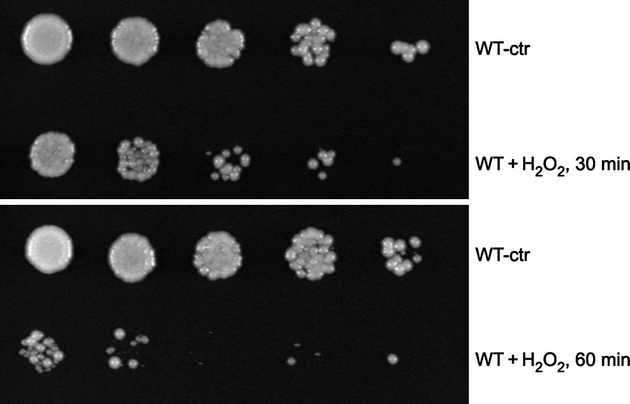
Spot assays showing H_2_O_2_-caused cell death. A fivefold serial dilution of cells from the wild type (WT) with or without (ctr) the addition H_2_O_2_ was spotted onto YPD plates growing for 3 days at 30 °C.

Because of the antioxidant nature of glutathione, we asked whether oxidative stress will alter the abundance and distribution of glutathione. Oxidative stress was achieved by direct addition to the culture medium of 5 mM H_2_O_2_ for 60 min, which causes apoptotic cell death (Madeo *et al*., 1999). The treatment of H_2_O_2_ strongly influenced glutathione labeling in the cells. Three types of cells with different glutathione status were observed after H_2_O_2_ treatment (Table 1, Figs 4 and 5): (I) cells with higher labeling intensity than wild type without H_2_O_2_ treatment in all cell compartments (Fig. 4b,f and 5); (II) cells with higher labeling intensity than wild type without H_2_O_2_ treatment in all cell compartments, except mitochondria, where a decreased level of labeling was observed (Fig. 4c,g and 5); (III) cells with decreased global glutathione labeling compared with wild type (Fig. 4d,h and 5). The most significant decrease in labeling intensity in cell type III was found in mitochondria, about 87% decrease compared with the wild type (Fig. 5). Enlarged/swollen mitochondria, chromatin condensation in nuclei, and increased number of vacuoles were also observed in cell type III (Fig. 4d,h and 6c,d), suggesting that these cells were undergoing apoptotic cell death. In opposite to cell type III, the ultrastructure of cell types I and II remained unaffected and appeared similar as the control (Fig. 6b). About 45% of the total cell population was type III cells, similar to the cell death rate when treated with 5 mM H_2_O_2_ reported previously (Yang *et al*., 2008) and in this study by spot assays (Fig. 7). We speculate that the increased level of glutathione in cell types I and II is a protection mechanism of yeast cells against the externally imposed oxidative stress. One evidence of this speculation is the high labeling intensity of cell wall (Fig. 5), where the external stress was first encountered and where the defense started. The labeling intensity was almost 1.5 times higher in cell type I compared with wild type (Table 1). As the defense moves on, glutathione is gradually consumed, especially in mitochondria (cell type II), and ultimately triggers the apoptotic cell death (cell type III).

**Table 1:** Average amounts of gold particles bound to glutathione per μm^2^ in whole cells of *Saccharomyces cerevisiae*. Cells were grown on YPD and SC^−GSH^ media and treated with or without hydrogen peroxide (H_2_O_2_, control)

	YPD Control	YPD H_2_O_2_	SC^−GSH^ Control	SC^−GSH^ H_2_O_2_	YPD *gsh1Δ*	YPD *gsh2Δ*
	118 (± 7)^d^		116 (± 8)^d^		12.6 (± 1)^h^	23.7 (± 2)^g^
Cell type I		228 (± 12)^a^		186 (± 8)^b^		
Cell type II		194 (± 8)^b^		153 (± 12)^c^		
Cell type III		76 (± 5)^e^		61 (± 5)^f^		

Values are means and SE. Significant differences between the samples are indicated by different lower case letters; samples that are significantly different from each other have no letter in common. *P*< 0.05 was regarded significant, analyzed by the Kruskal–Wallis test, and followed by *post hoc* comparison according to Conover. *n* ≥ 50 cells per growth medium.

### Glutathione contents in the intra- and extracellular fluids

**Fig. 8:**
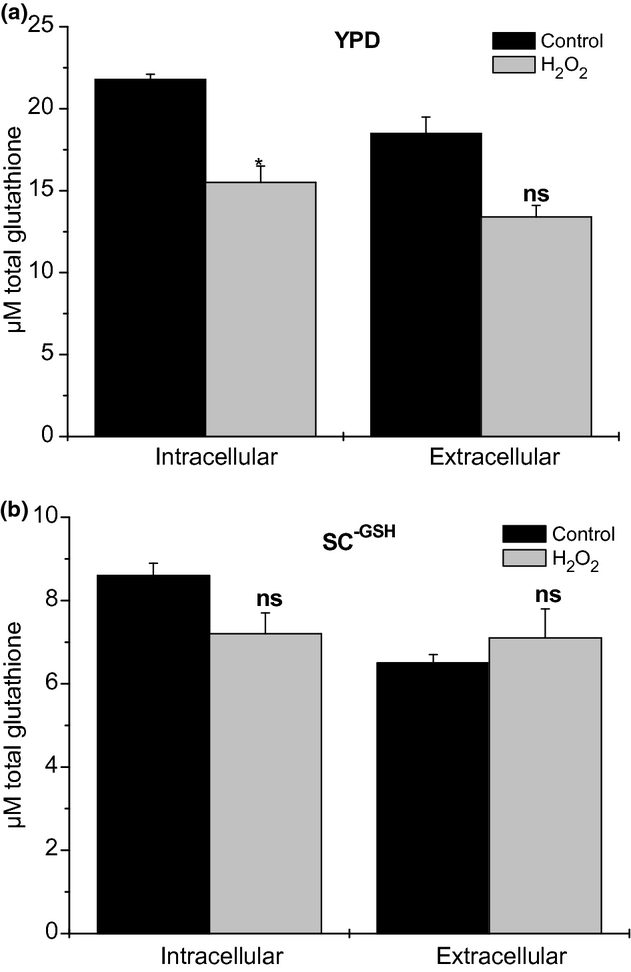
Total glutathione contents measured in the intra- and extracellular fluids. Graphs show mean values with SE of glutathione contents (total glutathione contents in μM) in the intra- and extracellular fluids of *Saccharomyces cerevisiae* grown on YPD and SC^−GSH^ media with and without the addition of 5 mM H_2_O_2_ for 60 min. Significant differences were calculated using the Mann–Whitney *U*-test; *significance at the 0.05 level of confidence (ns = not significant different), *n* = 3.

Glutathione contents were in general higher in the intracellular fluid of control cells grown on YPD and SC^−GSH^ where they reached 21.8 and 8.6 μM, respectively, than in the extracellular fluid where the concentrations of glutathione were found to be 18.5 and 6.5 μM (Fig. 8). H_2_O_2_ treatment significantly reduced glutathione contents in the intracellular fluid (29%) of cells grown on YPD medium. A decrease of 27% was observed in the extracellular fluid of these cells (Fig. 8). Even though glutathione contents were slightly decreased (16%) in the intracellular fluid of cells grown on SC^−GSH^ and increased in the extracellular fluid (9%) after H_2_O_2_ treatment, both changes were statistically insignificant (Fig. 8).

### Determination of cell survival rates and cell death

**Fig. 9:**
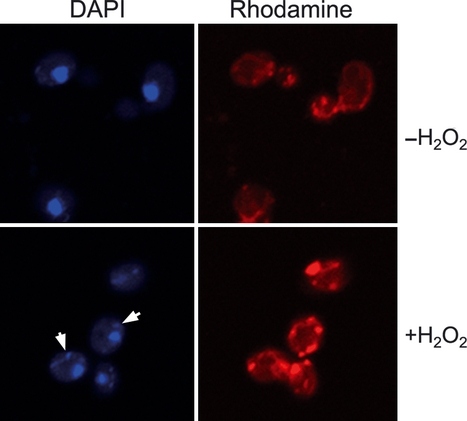
DAPI and rhodamine-phalloidin staining showing changes in nuclear and actin structure upon treatment of H_2_O_2_. Nuclear breakdown is visualized by small fragments of nuclei inside the cells (arrows), and actin destruction is indicated by the formation of large actin clumps.

Spot assay showed that H_2_O_2_ treatment dramatically decreased the cell survival rate. About 30–70% cells died when they were treated with H_2_O_2_ for 30 or 60 min (Fig. 7). We further analyzed the cell death using DAPI and rhodamine-phalloidin staining. DAPI staining showed that H_2_O_2_ treatment for 60 min caused nuclear breakdown, an indicator of apoptotic cell death. The apoptotic cell death was also revealed by rhodamine-phalloidin staining, which stains actin structure. As shown in Fig. 9, actin in untreated cells is primarily localized on cell periphery and forms filamentous structure. Actin in cells treated with H_2_O_2_ for 60 min however forms big clumps, indicating the destructed cytoskeleton.

## Discussion

The subcellular distribution of glutathione was studied in cells of *S. cerevisiae* by quantitative immunoelectron microscopy. The accuracy of glutathione-specific labeling was supported by the data (absence of labeling) obtained from preadsorption experiments of glutathione antisera with GSH and GSSG as well as by the lack of labeling on cells, which were treated with a preimmune serum instead of the primary antibody against glutathione. Additionally, knockout mutants of either *GSH1* or *GSH2*, which are both essential enzymes for the production of glutathione (Grant, 2001), showed a strong decrease in glutathione-specific labeling. The remaining labeling observed within the *gsh1Δ* and *gsh2Δ* was due to the fact that the YPD growth medium contains GSH, which is essential for proper development and growth of *S. cerevisiae* (Lee *et al*., 2001; Ayer *et al*., 2010). The mutants did not grow on SC^−GSH^. Thus, these results also reflect that *S. cerevisiae* is able to obtain and withdraw glutathione from the growth medium when internal concentrations are too low for growth and development (e.g. *GSH1* and *GSH2* knockout mutants). Transport of glutathione through the plasma membrane of yeast cells can be facilitated through transporters of the OPT family. The high-affinity transporters for glutathione, Hgt1p and Pgt1, have been localized in the plasma membrane of *S. cerevisiae* and *Schizosaccharomyces pombe*, respectively, (Bourbouloux *et al*., 2000; Thakur *et al*., 2007; Kaur *et al*., 2009) and are responsible for the uptake of glutathione from the growth media.

Within cells of *S. cerevisiae*, glutathione was found in different compartment-specific concentrations. The highest glutathione-specific labeling was found in mitochondria and was two to three times higher than in the cytosol of cells grown on YPD and SC^−GSH^ medium, respectively. These results are similar to what has been found in plant and animal cells (Huster *et al*., 1998; Zechmann & Müller, 2010), and they demonstrate the importance of high glutathione pools in mitochondria for proper cell development and growth in eukaryotes. The *GSH1* and *GSH2* knockout mutants showed a decrease in glutathione in mitochondria between 64% and 66%, whereas a much stronger decrease between 82% and 94% was found in all other cell compartments inside these cells. It has been demonstrated in a recent study that under conditions of GSH starvation, the redox environment of mitochondria from the same *GSH1* knockout mutant remained in more reduced state when compared to the cytosol (Ayer *et al*., 2010). These results indicate that in situations of glutathione starvation, glutathione contents in mitochondria preferentially accumulate in the form of GSH at the expense of glutathione pools in the cytosol. Additionally, it was demonstrated that the *GSH1* knockout mutant suffers irreversible respiratory incompetency and after five divisions, the majority of cells lack mitochondrial DNA when grown without GSH (Ayer *et al*., 2010). Thus, it seems that high levels of glutathione in mitochondria, preferentially in the form of GSH, are essential for proper cell growth and eventually, the survival of yeast cells. Similar conclusions were also drawn from studies with the glutathione-deficient *rml1* and *pad2-1* mutants (Zechmann *et al*., 2008; Zechmann & Müller, 2010). Even though both plants show a strong reduction of glutathione contents in all cell compartments when compared to the wild type, only the *rml1* mutant develops a severe phenotype such as the lack of a root meristem, short shoots, small rosettes, inflorescence, or flowers (Cheng *et al*., 1995; Vernoux *et al*., 2000; Cairns *et al*., 2006). The severe growth defects of the *rml1* mutant could be correlated with decreased levels of glutathione also in mitochondria (−97%). The unaffected phenotype of the *pad2-1* mutant could be correlated with control levels of glutathione in mitochondria despite a strong decrease in glutathione of up to 90% in all other cell compartments (Zechmann *et al*., 2008; Zechmann & Müller, 2010).

In this study, glutathione was similarly distributed in the cytosol and nuclei in yeast cells. These results could be an indication that nuclear membrane/nuclear pores are permeable to glutathione, which would result in similar glutathione concentrations between these two cell compartments. Within the nucleus, glutathione could serve as a regulator of nuclear proteins, control the nuclear redox potential, protect nuclear proteins against oxidation by glutathionylation and have important signaling functions (Vivancos *et al*., 2010).

Glutathione-specific labeling was also observed in cell walls and vacuoles, both containing very low concentrations in comparison with the other cell compartments. Glutathione was detected in cell walls of cells grown on SC^−GSH^ indicating that glutathione may actually be released into cell walls, which could prove an interesting aspect for commercial glutathione production (Rollini *et al*., 2010). These results were different from what was found in cyanobacteria and plants where glutathione was not detected inside cell walls (Zechmann & Müller, 2010; Zechmann *et al*., 2010). The functions of glutathione in cell walls of *S. cerevisiae* still have to be established, but they could include the defense against extracellular ROS and the detoxification of heavy metals in the extracellular fluid. Cells grown on YPD medium contained more than four times the amount of glutathione-specific labeling in vacuoles than cells grown on SC^−GSH^, demonstrating that glutathione is relocated and stored in vacuoles in situations of high internal glutathione concentrations. In *S. cerevisiae*, similar effects were only found during nitrogen depletion (Mehdi & Penninckx, 1997; Mehdi *et al*., 2001) and cadmium exposure (Adamis *et al*., 2007). In both cases, transporters of the ABCC family (Ycf1p and Bpt1p) were identified as being the main transport system of glutathione through the tonoplast into the vacuole (Mehdi *et al*., 2001; Paumi *et al*., 2009). In plants, a similar accumulation of glutathione (mainly in the form of GSSG) was observed during unnaturally high intracellular H_2_O_2_ production, associated with a strong increase in glutathione contents (Queval *et al*., 2011), and during sulfur treatment correlated with high amounts of internal glutathione content (Höller *et al*., 2010). Thus, it appears that the relocation and storage of glutathione in vacuoles can be used for the detoxification of toxic components such as cadmium, the supply of amino acids during nitrogen starvation, and during situations of high internal glutathione concentrations.

The induction of oxidative stress by H_2_O_2_ treatment had significant effects on glutathione distribution. Glutathione contents in the intracellular fluid decreased after H_2_O_2_ treatment. Nevertheless, this decrease did not induce a significant accumulation of glutathione in the extracellular fluid, indicating that oxidative stress diminishes glutathione inside the cell rather than causing an accumulation of glutathione in the extracellular fluid. Three different cell types based on gold particle density were distinguished. Cell type I showed a massive increase in glutathione in all cell compartments inside the cell, which likely reflected the immediate response of *S. cerevisiae* to oxidative stress. The strongest increase was found in mitochondria. Cell type II contained higher glutathione contents in all cell compartments inside the cell except mitochondria. These cells showed a similar ultrastructure to that of the untreated wild type. We speculate that the decrease in the mitochondrial glutathione is because of the externally exposed oxidative stress. Cell type III showed decreased levels of glutathione in all cell compartments inside the cells with the strongest depletion in mitochondria. This cell type represents cells that went through oxidative stress–induced apoptosis. In this study, typical ultrastructural alterations, as described previously (Ink *et al*., 1997), such as condensed chromatin, nuclear breakdown, increased vacuolation, swollen mitochondria, and cytoskeleton destruction, could be observed with both transmission electron and light microscopy. None of the above-mentioned cell types are comparable to quiescent yeast cells in stationary phase, as they contained clearly visible mitochondria and ER, which are not present in quiescent cells (Allen *et al*., 2006). Additionally, the observed cell types do not represent nonquiescent cells in the stationary phase. Cell types I and II do not show any signs of autophagy, and cell type III shows advanced apoptosis, which differs significantly from the described ultrastructure of nonquiescent cells in the stationary phase (Allen *et al*., 2006). On the subcellular level, several interesting observations were made during H_2_O_2_ treatment. Vacuoles showed a massive increase in glutathione-specific labeling in cell type III. A similar accumulation of glutathione in the form of GSSG could be observed in plant cells during increased intracellular H_2_O_2_ production (Queval *et al*., 2011) as described earlier. Thus, it seems probable that the observed labeling in *S. cerevisiae* during H_2_O_2_ treatment represented GSSG rather than GSH. The sequestration of GSSG in the vacuole may function to maintain a reduced environment in the cytosol, which would protect the cell against a shift toward an excessive, positive redox potential or a more oxidized state (Tommasini *et al*., 1993; Queval *et al*., 2011).

The results of this study also emphasize important roles of glutathione in mitochondria and the ER during oxidative stress. Mitochondria showed the strongest increase in glutathione (125%) of all compartments inside the cells of cell type I. Additionally, mitochondria showed the strongest decrease in glutathione-specific labeling in cell types II and III of all cell compartments. In cell type II, particularly, mitochondria were the only cell compartment that showed a decrease in glutathione-specific labeling when compared to the control. A drop in glutathione contents in mitochondria during H_2_O_2_ treatment could induce the activation of apoptosis as it could lead to increased amounts of ROS that can induce the expression of apoptosis inducing factors located in mitochondria (Perrone *et al*., 2008). The importance of high glutathione levels in mitochondria is also supported by the observation that *GSH1* knockout mutants suffer irreversible respiratory incompetency and after five divisions, the majority of cells lack mitochondrial DNA when grown without GSH (Ayer *et al*., 2010). These results could be correlated with a strong decrease in glutathione levels in the mitochondria (about −60%) of the *GSH1* knockout mutant in this study. Glutathione-specific labeling was also observed along and inside the membranes of the ER, which was similar to the situation in plants (Zechmann *et al*., 2008; Zechmann & Müller, 2010). GSH at the ER plays essential roles for proper folding of glyco- and secretory proteins, which is based on the formation of native disulfide bonds within the ER lumen by maintaining ER oxidoreductases in a reduced state (Jessop & Bulleid, 2004; Perrone *et al*., 2008). This is especially crucial during situations of oxidative stress when large amounts of GSSG are produced inside the cell. ER stress, elevated ROS, and changes in the redox state could be correlated with the appearance of some programmed cell death markers, whereas the supplementation of glutathione led to decreased ROS production and increased cell survival (Haynes *et al*., 2004).

In summary, this study gives a detailed insight into the subcellular distribution of glutathione in *S. cerevisiae*. Mitochondria were found to be the compartment of highest glutathione accumulation, followed by the cytosol and nuclei with the lowest levels in vacuoles and cell walls. As a decrease in glutathione content in mitochondria could be correlated with oxidative stress and apoptosis, it seems that high levels of glutathione in mitochondria play an important role for the development, growth, and defense against ROS. Additionally, the importance of vacuoles for the sequestration of glutathione (most probably GSSG) could be identified as an important defense mechanism for the protection of cells against ROS. The observation of glutathione-specific labeling in cell walls opens the question about its functions in the defense against extracellular stress, which has to be resolved in future studies. The method presented in this study will help clarify the compartment-specific importance of glutathione in *S. cerevisiae* during cell growth, development, and defense and will contribute toward a better understanding of compartment-specific glutathione metabolism during situations of oxidative stress.

## Statement

Re-use of this article is permitted in accordance with the Terms and Conditions set out at http://wileyonlinelibrary.com/onlineopen#OnlineOpen_Terms
